# Monitoring and Measurement of Intracranial Pressure in Pediatric Head Trauma

**DOI:** 10.3389/fneur.2019.01376

**Published:** 2020-01-14

**Authors:** Sarah Hornshøj Pedersen, Alexander Lilja-Cyron, Ramona Astrand, Marianne Juhler

**Affiliations:** ^1^Department of Neurosurgery, Copenhagen University Hospital, Copenhagen, Denmark; ^2^Department of Neurosurgery, Aarhus University Hospital, Aarhus, Denmark

**Keywords:** intracranial pressure (ICP), age-dependent, reference values, traumatic brain injury (TBI), head trauma, children, pediatric, guidelines

## Abstract

**Purpose of Review:** Monitoring of intracranial pressure (ICP) is an important and integrated part of the treatment algorithm for children with severe traumatic brain injury (TBI). Guidelines often recommend ICP monitoring with a treatment threshold of 20 mmHg. This focused review discusses; (1) different ICP technologies and how ICP should be monitored in pediatric patients with severe TBI, (2) existing evidence behind guideline recommendations, and (3) how we could move forward to increase knowledge about normal ICP in children to support treatment decisions.

**Summary:** Current reference values for normal ICP in adults lie between 7 and 15 mmHg. Recent studies conducted in “pseudonormal” adults, however, suggest a normal range below this level where ICP is highly dependent on body posture and decreases to negative values in sitting and standing position. Despite obvious physiological differences between children and adults, no age or body size related reference values exist for normal ICP in children. Recent guidelines for treatment of severe TBI in pediatric patients recommend ICP monitoring to guide treatment of intracranial hypertension. Decision on ICP monitoring modalities are based on local standards, the individual case, and the clinician's choice. The recommended treatment threshold is 20 mmHg for a duration of 5 min. Both prospective and retrospective observational studies applying different thresholds and treatment strategies for intracranial hypertension were included to support this recommendation. While some studies suggest improved outcome related to ICP monitoring (lower rate of mortality and severe disability), most studies identify high ICP as a marker of worse outcome. Only one study applied age-differentiated thresholds, but this study did not evaluate the effect of these different thresholds on outcome. The quality of evidence behind ICP monitoring and treatment thresholds in severe pediatric TBI is low and treatment can potentially be improved by knowledge about normal ICP from observational studies in healthy children and cohorts of pediatric “pseudonormal” patients expected to have normal ICP. Acceptable levels of ICP − and thus also treatment thresholds—probably vary with age, disease and whether the patient has intact cerebral autoregulation. Future treatment algorithms should reflect these differences and be more personalized and dynamic.

## Introduction

Traumatic brain injury (TBI) is one of the leading causes of mortality among children and adolescents, and a great contributor to morbidity (1, 2). The annual incidence of reported TBI cases per 100.000 people (due to all causes) is higher in high-income countries than in low—and middle-income countries (3), with an annual incidence of children with a TBI related emergency department visit estimated to 691 per 100.000, hospitalization due to TBI to 74 per 100.000 and TBI related death to 9 per 100.000 (4). These numbers may also reflect differences in reference and reporting patterns in different geographical areas (5, 6). However, the total burden of TBI cases are nearly three times higher in low-income countries, with road traffic accidents being the leading cause (3). The risk of road traffic deaths in low-income countries are by WHO reported three times higher than in high-income countries, and the leading cause of all deaths in age group 5–29 years (7).

Although the number of pediatric patients sustaining a severe TBI is increasing, the understanding of pathophysiology and long-term outcome remains limited. Most clinicians argue that therapy strategies should be based on high-quality research, conducted either as randomized clinical trials (RCT) or observational studies with high-quality body of evidence. Where an RCT aims to eliminate as many confounding variables as possible, a high-quality observational study aims to clarify those variables. In the last decades, only ten RCTs in pediatric patients with severe TBI have been conducted and the level of evidence in observational studies is reported as low or moderate (8–10). This affects both international guidelines for management of severe pediatric TBI and treatment algorithms at individual TBI centers. A survey from 2013 conducted at 32 American and European pediatric TBI centers revealed high variability in treatment algorithms, particularly for topics with limited evidence (11). However, both monitoring of intracranial pressure (ICP) and treatment of intracranial hypertension were an integral part of TBI management despite the lack of evidence, and all centers unanimously reported the use of an ICP threshold of 20 mmHg. Eight centers further reported age-specific ICP threshold values with slightly lower values in younger patients (10 mmHg at one center, 15 mmHg at four centers and 18 mmHg at three centers) (11).

In this focused review we discuss the use of different ICP monitoring modalities in the treatment of pediatric TBI. Furthermore, the existing evidence behind the Brain Trauma Foundation guidelines for ICP (10) are evaluated, and it is discussed how ICP treatment in severe pediatric TBI can potentially be improved by improved knowledge about normal ICP in children and age-specific threshold values.

## ICP Monitoring Technology

The first data on invasive measurement of ICP were published by Guillaume and Janny in 1951 (12), and the first comprehensive analysis of ICP curve morphology was performed in patients with probable space occupying lesions by Lundberg in 1960 (13) and in patients with TBI in 1965 (14). The measurements were obtained through a transducer coupled to an external ventricular drain (EVD). Today, in continuous monitoring of ICP in patients admitted to neuro-intensive care unit, ICP is often measured using a parenchymal sensor. In infants, there are two additional possibilities to indirectly evaluate ICP; (1) by palpating the open anterior fontanelle and cranial sutures, and (2) by serial measurements of head circumference. Although palpation of the anterior fontanelle can be used for screening of patients for further investigations, neither palpation nor head circumference are used in management of acute severe pediatric TBI (15). Other non-invasive methods of ICP estimation (e.g., contrast-enhanced ultrasonography, magnetic resonance imaging, near-infrared spectroscopy, optic nerve sheath diameter, otoacoustic emission, quantitative pupillometry, transcranial doopler) are constantly being improved, but have not yet achieved quantitation of absolute ICP values or reached a level of accuracy sufficient for treatment decisions in clinical practice (16–19).

### Measurement of ICP Through an External Ventricular Drain

**Figure 1:**
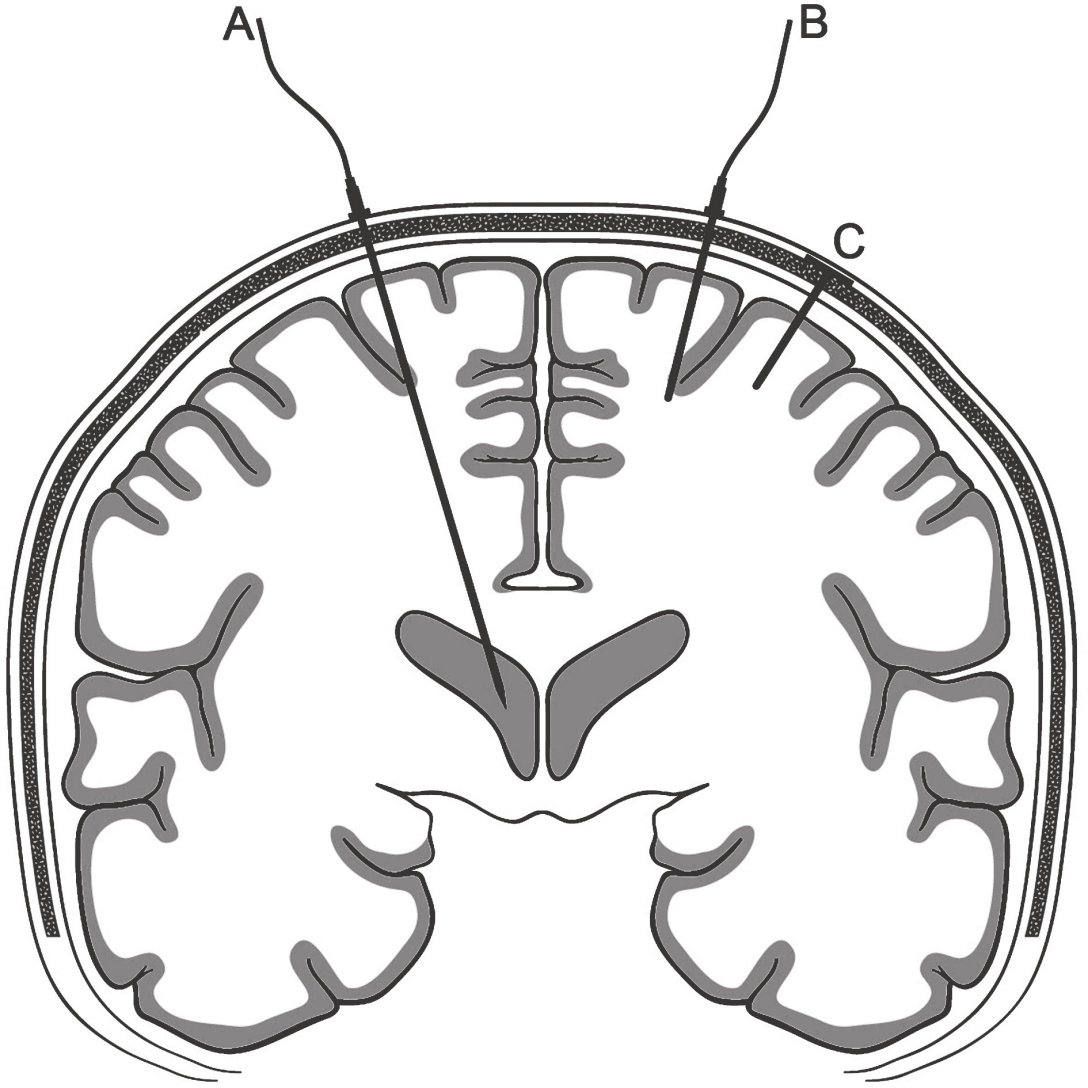
ICP monitoring technologies. The figure is a schematic drawing of different ICP monitoring devices in a coronal plane. The placement of an EVD (corresponding to Kocher's point) **(A)**, a parenchymal ICP sensor **(B)**, and a telemetric ICP sensor **(C)** are shown.

The gold standard to measure ICP is through an EVD coupled to an external fluid-filled transducer (Figure 1A) with the draining end closed for an exact ICP measurement (17). An EVD is often placed at the non-dominant side through a burr hole at Kocher's point. No recommendations on drain placement exits, if the patient has focal lesions in the non-dominant hemisphere.

The overall complication rate to EVD treatment in the pediatric population is around 20–25% including infection, misplacement, hemorrhage, and malfunction (occlusion with cellular debris or collapse of the ventricular system around the drain tip) (20). The most common complication is infection, estimated to occur in around 10% of the patients (20, 21), which is comparable to the rate in adult populations (22). The rate of EVD related infections may be lowered using prophylactic antibiotics, including antimicrobial impregnated catheters, although this might increase the rate of infections with more resistant bacteria, such as methicillin resistant Staphylococcus Aureus (23, 24). Secondly, the pediatric patient often has a very narrow ventricular system, which makes the placement of the ventricular catheter difficult and may increase the risk of malfunction. Correct placement can be aided by guide (e.g., the Ghajar guide or the Thomale guide) (25, 26), surgical navigation (27) and maybe in the future, holographic visualization of the ventricular system (28). Finally, placement of the external transducer/choice of reference point strongly influences measurement levels and is a source of potential error. EVDs with integrated ICP sensors at the tip inside the ventricular system (Raumedic Neurovent) or in the parenchyma (Spiegelberg ventricular probe) eliminate this source of error. In addition, these devices also allow both drainage and continuous ICP measurements.

### Measurement of ICP Using a Parenchymal ICP Sensor

Several parenchymal ICP monitoring devices exist, using different technologies including fiber optic sensors (e.g., Camino ICP Monitor), strain gauge devices (e.g., Codman MicroSensor and Raumedic Neurovent-P ICP sensor) and pneumatic sensors (Spiegelberg) (Figure 1B) (17). A parenchymal ICP sensor is often placed in the non-dominant frontal region. The placement can be modified if focal lesions are verified or suspected. There is, however, no consensus whether “true” ICP is measured in the healthy hemisphere or the damaged hemisphere. An interhemispheric supratentorial pressure gradient in patients with head trauma and focal lesions has been documented suggesting that such patients could benefit from bilateral ICP monitors (29). However, in a setup with bilateral measurement a concern would be the risk of a pressure gradient between the two sensors due to technical issues and not resulting from biological causes (30, 31). Other sensor locations aside from the brain parenchyma such as the subdural or epidural spaces have been investigated, but are less used in daily clinical practice (32–35).

The complications using parenchymal sensors are infection and hemorrhage (17). Technical errors, with a particular risk of baseline-drift with time, might be especially relevant in the neuro-intensive care setting due to the frequent occurrence of electrostatic discharges (30). Such baseline-drifts can be sudden (“baseline-shifts”) or gradual and can sometimes be identified by a discrepancy between the pulse wave amplitude and the ICP value, as the amplitude will increase parallel to increasing ICP. A review comparing technical aspects and complication rate of the different sensor types was published in 2012 (17).

For nearly a decade, telemetric ICP monitoring has been possible through the Raumedic Neurovent-P-tel, which is a parenchymal strain gauge sensor coupled to a wireless transcutaneous data transmitter (Figure 1C) (36). So far, telemetric ICP monitoring has been applied only in severe adult TBI (37). In previous investigations (38, 39), complication rates were similar to those of cabled ICP sensors (40–42). Another telemetric device (Miethke/Aesculap Sensor Reservoir) has also been developed to measure ICP through an implanted ventricular shunt system (43, 44). In principle, this could also be coupled to an EVD, but so far there are no reports testing the device in a neuro-intensive care setting.

### Comparison of The Different Techniques

The Brain Trauma Foundation guidelines recommend the use of ICP monitoring to determine if intracranial hypertension is present, while drainage of cerebrospinal fluid (CSF) through an EVD is suggested to manage intracranial hypertension (45). The decisions on how to monitor ICP and where to monitor ICP are still based on local standards, the individual case, and the clinician's choice.

ICP monitoring through an EVD provides the possibility to perform intermittent or continuous ICP measurement as well as therapeutic interventions such as treatment of elevated ICP through drainage of CSF, and intrathecal administration of medicine (e.g., antibiotics) (17). Another advantage of ICP measurement through an EVD is the possibility to directly measure water column height and recalibrate the transducer, which is not possible for most parenchymal ICP sensors (except for the Spiegelberg sensor). Lack of recalibration can cause a risk of treatment decisions made on incorrect ICP values. In a recently published systematic review, no differences in mortality or functional outcome in patients with TBI could be detected comparing ICP measurement through an EVD to a parenchymal sensor. The overall complication rate was, however, higher in EVDs, mainly due to infections (46).

In summary, both measurement sites (intraventricular vs. parenchymal) have advantages in clinical decision making in children with severe TBI. Though the parenchymal ICP sensors have equal accuracy and probably a slightly lower complication rate compared to intraventricular ICP monitoring, the latter remains gold standard (41, 42, 47–49). This may be explained by; (1) a historical perspective, (2) a less significant intercompartment pressure gradient, and (3) validation of measured ICP through an external fluid column (23).

## ICP in Children

**Figure 2:**
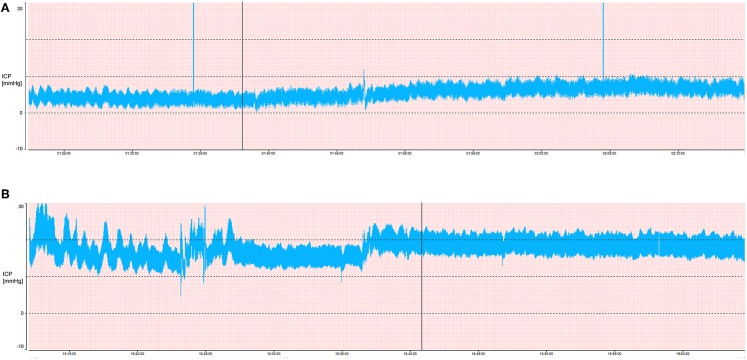
ICP and pulse wave amplitude. The two pressure curves illustrate ICP tracings at mean ICP 5 mmHg **(A)** and mean ICP 20 mmHg **(B)**. At 20 mmHg (recommended treatment threshold), the amplitude is higher consistent with increased pulsatility and decreased compliance. The probability of abnormal ICP patterns (A waves and tall B waves) also occur frequently ≥20 mmHg, but are not seen at 5 mmHg, where the signal is much more uniform and stable.

**Figure 3:**
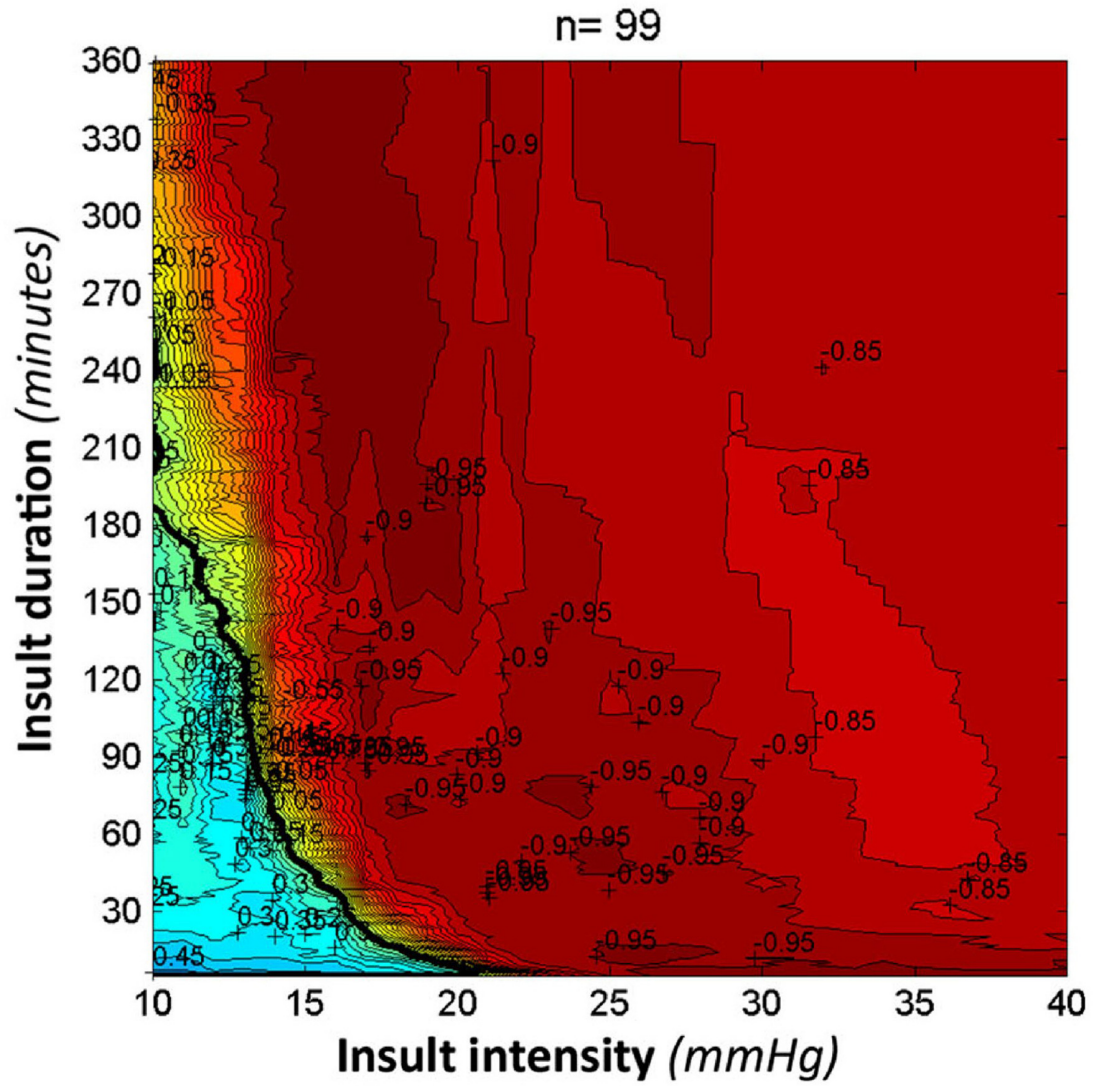
ICP tolerance. This figure was adapted and used with permission from Intensive Care Medicine Journal. It visualizes the correlation between outcome on the Glasgow Outcome Scale and the time burden of intracranial hypertension in a pediatric population with traumatic brain injury. Red episodes illustrate a low score on the Glasgow Outcome Scale (worse outcome), while blue episodes illustrate a high score (good outcome). The black curve is named the transition curve and illustrates zero correlation (51).

ICP treatment in TBI aims at reducing an elevated ICP in order to improve outcome. The treatment threshold is 20 mmHg in children and 22 mmHg in adults (10, 50). However, it can be questioned how close the current threshold is to normal ICP (Figure 2). Güiza et al. (51) showed that outcome measured using the Glasgow Outcome Scale in patients with TBI depends on the cumulated duration of episodes with elevated ICP and that the tolerated burden is less in children than in adults. The tolerance for ICP > 20 mmHg is only 7 min in children (vs. 37 min in adults), and for an ICP of 10 mmHg it is 180 min (Figure 3). As the tolerance for normal ICP levels should be indefinite, this could indicate that normal ICP in children is <10 mmHg. However, if cerebral autoregulation is intact, the tolerance level for ‘indefinite duration’ is shifted to 15 mmHg (51).

### Normal ICP Reference Values

Obtaining reliable, quantitative ICP values still involves performing invasive intracranial measurements, which is the straightforward explanation behind the lack of reference values for normal ICP. Currently, ICP in “pseudonormal” subjects; i.e., patients in whom ICP/CSF related pathology is absent or unlikely, provides an insight into ICP ranges which are probably normal. This kind of documentation indicates that normal ICP is considerably lower than previously assumed and strongly dependent on postural changes. Values obtained in this way in adults range between approximately 0 to 5 mmHg in supine position and −5 to 0 mmHg in upright position (52, 53). Interestingly, Lundberg's ground-breaking work from 1960 included just one patient, who was retrospectively considered to have normal ICP, and in whom the supine intraventricular pressure recorded continuously was around 0 mmHg (13). In children, the evidence for normal ICP values is even more scarce, and most studies are conducted in children with TBI, cranial synostosis or shunt-managed hydrocephalus, i.e., situations from which normal ICP cannot be extrapolated. In a series on shunted pediatric patients, the ICP range in children with functional shunts (neither under nor overdrainage) was −1.6 to 16.9 mmHg, but the range overlapped the overdrainage group (54), and as shunt treatment directly affects ICP, normal ICP levels cannot be inferred from shunted cohorts even if ICP is “well-managed”.

We have examined a “pseudonormal” mixed pediatric and adult cohort undergoing ICP monitoring which was considered normal, and in whom there was no further suspicion of increased ICP or need for pressure relieving treatment during a minimum follow-up period of 3 years following the measurement (55). Mean daytime ICP in children was 2.8 mmHg ± 2.2 vs. 1.9 mmHg ± 4.2 in adults. Mean night-time ICP was 6 mmHg higher in both children and adults. Surprisingly, this study also showed an inverse relationship between age and ICP with a decrement of 1 mmHg per decade. This is in obvious disagreement with the generally accepted perception that ICP is lower in children than in adults. However, the same age-related ICP pattern was shown in a mixed diagnostic cohort from age 16 to 85 years (56). Studies examining the lumbar puncture opening pressure (CSF_op_) specify a diagnostic cut-off at 25–28 cm H_2_O (18–21 mmHg) (57, 58). CSF_op_ could be an ethically more acceptable way of documenting truly normal ICP values, but there are limitations extrapolating these values to reference values for intracranially measured ICP. CSF_op_ is a momentary measurement and the body position necessary for performing the lumbar puncture will itself increase the measured value (52).

In summary, little is known about “normal” ICP in children and reference values are either extrapolated from adults or from pediatric patients in whom ICP must be considered abnormal. Since children differ from adults in both anatomy and physiology (59) reference values including treatment threshold in severe pediatric TBI should reflect this.

## Evidence Behind Guideline Recommendations Regarding ICP Monitoring and Treatment Threshold of ICP

ICP monitoring in pediatric TBI relates to the 10% who suffer a moderate or severe head trauma with a higher risk of intracranial complications (60). The updated guidelines provides recommendations for clinical decisions and treatment algorithms including evidence—and consensus-based suggestions for both first and second tier treatment (10, 45). Despite a systematic review of the literature, only low-quality studies and few moderate-quality studies have been found, leaving no level I recommendation, few level II recommendations and a majority of level III recommendations to guide the clinician.

### The Use of ICP Monitoring

A total of 19 studies examining if treatment decisions based on ICP monitoring improves outcome were included in the 3rd edition of the guidelines (10). Three large retrospective multicenter studies [one using hospitals as unit of measurement (61) and two using patients as unit of measurement (62, 63)] were added since the 2nd edition and provides evidence that ICP monitoring and treatment of increased ICP improves clinical outcome. Based on the included studies, the guidelines recommend ICP monitoring in severe pediatric TBI (level III recommendation) (10). However, it is noteworthy that the three studies do not provide a unanimous conclusion.

Alkhoury et al. (62) aimed to determine the effect of ICP monitoring on mortality in pediatric patients with severe TBI and found that ICP monitoring only reduced mortality in patients with a Glasgow Coma Scale (GCS) score of 3. The two groups (ICP monitor vs. no ICP monitor) were comparable in age, sex, GCS and Trauma and Injury Severity Score, but differed in Injury Severity Score (higher in the ICP monitoring group) and Revised Trauma Score (lower in the ICP monitoring group). Patients who underwent ICP monitoring were found to have longer hospital admissions, including a longer stay in the neuro-intensive care unit and more ventilator days. This could either indicate a selection bias with more severe injuries in patients with an ICP monitor (not explained in the paper), that ICP monitoring itself keeps the patient in the neuro-intensive care setting or an increased risk of complications following ICP monitoring and potentially aggressive pressure relieving treatment.

To assess whether hospital factors (e.g., trauma level center, patient admissions) and ICP monitoring are associated with outcome Bennet et al. (61) included pediatric TBI patients admitted to 31 centers. They reported that hospitals with higher patient volumes and pediatric trauma level I centers were more likely to use ICP monitoring and that a higher patient volume was associated with a more standardized ICP management and an overall better patient outcome. Conclusively ICP guided management results in a more favorable outcome; it is however also possible that hospitals with more patient admissions and standardized ICP management provide an overall better patient care.

A subsequent study by Bennet et al. (63) found no evidence that ICP monitoring in pediatric patients with severe TBI improved outcome. However, unlike the groups formed by Alkhoury et al. (62), the initial assessment of patients in the ICP monitoring group revealed poorer Injury Severity Scores, head Abbreviated Injury Scale scores and GCS scores and a higher risk of an intracranial hemorrhage. In accordance with Alkhoury et al., patients with an ICP monitor had longer hospital admissions and received more treatment to manage intracranial hypertension. Further they had higher odds of mortality, discharge to hospice and to receive either a tracheostomy or a gastrostomy tube. If patients receiving an ICP monitor had a more severe injury, such a selection bias could explain why no association was found between ICP monitoring and improved outcome.

In summary, the ambiguous conclusions can be a result of inadequate control for statistically confounding factors (e.g., severity of injuries, different treatment algorithms for insertion of an ICP monitor, different standards of patient care in different centers). Interestingly, the retrospective multicenter studies revealed that only 7.7% (62), 32.5% (63), and 55.0% (61) of the included patients underwent ICP monitoring, although the use of ICP monitoring has been suggested since the initial guidelines in 2003. In both 2012 and 2017 Bennet et al. reported a high inter-hospital variation in the use of ICP monitoring [14–83% (61) and 6-50% (63), respectively] and over a 10-year period (2001-2011) the rate of ICP monitoring was decreasing, seemingly in contrast to the initial guidelines (61).

### The Threshold for Treatment of Intracranial Hypertension

Treatment threshold for ICP in the pediatric patient is based on 12 retrospective and prospective studies examining target values for lowering ICP to improve clinical outcome (10). Most studies applied an ICP threshold of 20 mmHg and reported lower ICP values in patients with a favorable outcome compared to those with an unfavorable outcome (64–70). Few studies examined if different threshold values resulted in different outcome [respectively 14/20/30 mmHg (69) and 15/20 mmHg (71)]. ICP values > 20 mmHg were found to be associated with an unfavorable outcome (64, 67, 69, 70), but no difference in outcome across the different threshold values could be detected (69, 71). Two studies even applied thresholds of 35 and 40 mmHg and found, not surprisingly, that values higher than the applied threshold were associated with an unfavorable outcome (72, 73). Based on these findings, the guidelines suggest a treatment threshold of 20 mmHg for 5 min (level III recommendation) (10).

Though age itself does not affect outcome (74), the definition of childhood (due to differences in anatomy and physiology between infants, children and adolescents) is extremely important in comparison of pediatric patients (59). None of the included studies examine comparable patient populations. One study includes infants from age 0–24 months (71), while others exclude the youngest patients (66, 68, 70, 72, 73, 75). Furthermore, the definition of a pediatric patient varies from 1–12 years of age (66), 0–13 years of age (64), 3 months to 14 years of age (73), 0–15 years of age (67), 1 month to 16 years (70), 3 months to 16 years of age (72), to 17 years of age (68), 2.4 months to 18 years of age (75) and 0–19 years of age (65). Furthermore, only one of 12 studies applied age-specific treatment thresholds (15 mmHg at age 0–24 months, ICP > 18 mmHg at age 25–96 months and ICP > 20 mmHg at age 97–214 months). However, it was not examined if age-differentiated thresholds were correlated with improved outcome (64).

Even though the guideline committee speculates in individualized ICP management and lack of existing normal values for ICP, the same treatment threshold is recommended across all age-groups. Interestingly, threshold values for CPP are suggested to be age-dependent with lowest values in infants (10). A well-documented age-dependent blood pressure (84) and the correlation between ICP, CPP and mean arterial blood pressure (MAP) (CPP = MAP-ICP) is not further addressed.

In summary, the lack of consistence in age of childhood and the differing contribution of extracranial injuries, challenges the threshold-comparison and emphasizes the need for greater consistency in pediatric research. The currently used treatment threshold is considerably higher than ICP reference values proposed in studies examining “normal” ICP (52, 55, 56, 76, 77), which could be one of the reasons for the still ambiguous benefit of ICP monitoring and regulation in pediatric patients with severe TBI.

## Future Perspectives

High-quality research in ICP monitoring and regulation in severe pediatric TBI is still limited. Limitations may be due to the heterogeneity in pathology, patient populations (as previously mentioned), treatment algorithms including threshold values and sensitivity and specificity in outcome measurements (8). Further research must be conducted for future guidelines to provide level I or level II recommendations. Studies can still add evidence by examining smaller, but more homogenous patient groups (8), as such studies can also be collected into meta-analysis protocols. The multicenter cohort observational study SYNAPSE-ICU is being conducted with the aim to describe worldwide current practices of ICP monitoring and ICP management in neuro-intensive care setting; unfortunately, this study only enrolls patients >18 years (78). The ADAPT trial describes the correlation between outcome and treatment approaches and decisions for pediatric TBI already used in clinical practice, aiming to provide evidence for new level II recommendations (9). The observational cohort study includes 51 centers and approximately 1,000 study subjects. Few preliminary results have been published, but to our knowledge data form this study to guide management of intracranial hypertension are still awaiting.

It is important to remember that outcome is not affected by ICP monitoring in itself, but only by the clinical consequences and actions based on it, and ICP control alone does thus not necessary lead to a good outcome (71). ICP is only one component in a complex cerebral homeostasis, which also includes CPP, autoregulation, oxygenation, and preservation of metabolism/blood flow index. An intact cerebral autoregulation protects the brain from inadequate blood flow despite changes in CPP. TBI can however affect the autoregulation and autoregulation in pediatric patients with severe TBI are reported impaired in 29–50% of the patients (79, 80). Several surrogate measurements for cerebral autoregulation exist (81), one being the pressure reactivity index (PRx) first described in 1997 (82). The PRx is the Pearson correlation between the slow waves of ICP and MAP and can be used to determine the individual optimal CPP and thus the maintenance of an efficient autoregulation level (83). High PRx values (indicating an impaired autoregulation) have within recent years found to be associated with higher mortality/unfavorable outcome in pediatric TBI (80, 83). Multimodal neuromonitoring of pediatric TBI patients covering several of these physiological interactions would potentially improve clinical management and may facilitate a more individualized treatment strategy. Comprehensive guidelines thus must be based on complex physiological algorithms. However, a very basic and simple first line challenge is to provide truly normal pediatric ICP reference values.

Determining the normal reference range for ICP in healthy children requires a patient cohort with no suspicion of CSF pathology. Due to its invasive nature, it is not ethically acceptable to measure ICP intracranially in a healthy child. A normal reference range with good statistical confidence requires measurements in large numbers in different age groups, and can therefore only be obtained through non-invasive ICP measurements; alternatively by extrapolating data from measurements in “pseudonormal” patient populations. Since ICP is strongly affected by body posture, the ICP monitoring technology must allow the child a free range of motion during measurement. As discussed, non-invasive methods for ICP estimation are improving, but still lack accuracy and are not suitable for continuous monitoring during daily activities. The telemetric ICP sensor can be used in the neuro-intensive care unit and can be left implanted for 3 months permitting ICP monitoring sessions both during recovery and during follow-up with return of daily life activities. This facilitates a useful ICP monitoring technology which can be used in a “pseudonormal” population with an initial need of ICP measurement, and a subsequent complete cerebral recovery.

Due to human physiology and established age-dependent values in both CPP and MAP, it may be assumed that ICP is also affected by age and body growth. An RCT including age-defined subgroups with three different ICP threshold values applied in each group could clarify if threshold values should differ between age-groups. The pediatric patient cohort (age 0–18) could be divided into subgroups corresponding to physiological milestones (e.g., cranial suture closure, change in CSF production, change in general body growth rate), while applied treatment thresholds in each group could be 20, 15, and 10 mmHg (and thus corresponding to/lower than recommended values).

## Conclusion

ICP monitoring and treatment of intracranial hypertension is a central part of the Brain Trauma Foundation guidelines for management of severe pediatric TBI. Due to the heterogeneity in TBI pathology, variation in patient populations, treatment algorithms, and outcome measures between centers/studies, the clinician is left with no high-level recommendations to guide the treatment of a child with a severe head trauma. Specifically regarding ICP monitoring and ICP treatment thresholds, evidence of a normal ICP range in children is lacking. No studies have evaluated the effect of different treatment thresholds on outcome. We therefore recommend that normal ICP reference values for infants, children and adolescents and age-specific treatment thresholds are established through further studies.

## Author Contributions

SP have been responsible for the primary drafting and revision of the paper. All authors (SP, AL-C, RA, and MJ) have been contributing to the drafting and revising of the paper, giving their approval for publication and agree to be accountable for all aspects of the work.

### Conflict of Interest

The authors declare that the research was conducted in the absence of any commercial or financial relationships that could be construed as a potential conflict of interest.
